# Controlled Synthesis of Pt Nanowires with Ordered Large Mesopores for Methanol Oxidation Reaction

**DOI:** 10.1038/srep31440

**Published:** 2016-08-23

**Authors:** Chengwei Zhang, Lianbin Xu, Yushan Yan, Jianfeng Chen

**Affiliations:** 1 State Key Laboratory of Organic–Inorganic Composites, Beijing University of Chemical Technology, Beijing 100029, China; 2 Tianjin Key Laboratory of Materials Laminating Fabrication and Interface Control Technology, Hebei University of Technology, Tianjin 300130, China; 3 Department of Chemical and Biomolecular Engineering, University of Delaware, Newark, Delaware 19716, USA

## Abstract

Catalysts for methanol oxidation reaction (MOR) are at the heart of key green-energy fuel cell technology. Nanostructured Pt materials are the most popular and effective catalysts for MOR. Controlling the morphology and structure of Pt nanomaterials can provide opportunities to greatly increase their activity and stability. Ordered nanoporous Pt nanowires with controlled large mesopores (15, 30 and 45 nm) are facilely fabricated by chemical reduction deposition from dual templates using porous anodic aluminum oxide (AAO) membranes with silica nanospheres self-assembled in the channels. The prepared mesoporous Pt nanowires are highly active and stable electrocatalysts for MOR. The mesoporous Pt nanowires with 15 nm mesopores exhibit a large electrochemically active surface area (ECSA, 40.5 m^2^ g^−1^), a high mass activity (398 mA mg^−1^) and specific activity (0.98 mA cm^−2^), and a good *I*_*f*_/*I*_*b*_ ratio (1.15), better than the other mesoporous Pt nanowires and the commercial Pt black catalyst.

The direct methanol fuel cells (DMFCs) have attracted great attention as promising candidates for portable, transportation and mobile applications because of their high power densities and high energy-conversion efficiencies^1^,^2^. The anode, where methanol is oxidized to produce carbon dioxide, protons and electrons, is a key component of DMFCs^3^. Until now, nanostructured Pt materials have been recognized among the most active anode catalysts for methanol oxidation due to their high surface areas, leading to economical and effective utilization of the expensive Pt catalysts. However, intermediate species produced in the process of electro-oxidation of methanol can poison the Pt catalysts, which limits the wide spread commercialization of the DMFCs. Since the activity and stability of the nanostructured Pt materials are highly dependent on their surface structure, particle size and morphology, great efforts have been focused on developing various nanostructured Pt materials such as Pt nanoparticles^4^,^5^, Pt nanowires^6^,^7^, Pt nanotubes^8^,^9^, Pt nanospheres^10^,^11^, and mesoporous Pt^12^,^13^ to achieve the Pt catalysts with higher activity and better poisoning tolerance. Among them, one-dimensional (1D) nanostructured Pt materials, such as Pt nanowires^6^,^7^ and Pt nanotubes^9^,^14^, have received growing attention due to their anisotropic morphology that can improve the mass and electron transport, and the catalyst utilization^15^. Furthermore, compared to 0D Pt nanoparticles, 1D Pt nanostructures have a better durability due to their longitudinal axis structures, which make the materials less vulnerable to dissolution and aggregation during the electrocatalytic electrode reactions^15^. However, 1D nanostructured Pt materials usually have smaller specific surface areas, resulting in inferior electrocatalytic activities to the Pt catalysts. Ordered mesoporous Pt nanowires, a novel type of 1D Pt nanomaterials, possess particular advantages of both 1D nanostructure and high surface area. Compared with the solid Pt nanowires, mesoporous Pt nanowires with ordered mesopores have larger specific surface area and are more easily accessible for the guest molecules due to their ordered interconnected mesopores^16^.

Typically, ordered porous metal nanowires can be prepared by a dual templating strategy which either combines a hard-templating (e.g., 1D porous membrane) and a soft-templating (e.g., lyotropic liquid crystal (LLC)) techniques (referred to as hard/soft-templating method) or employs both hard-templating (e.g., 1D porous membrane and self-assembled silica or polymer spheres) techniques (referred to as hard/hard-templating method). The dual templating method provides a satisfactory way to control the pore size and the surface structure of the wires. The diameter of the porous metal wires can be confined by the channels of the porous membrane and the pore structure can be controlled by the mesoporous template (such as LLCs as a soft template^17^,^18^,^19^, or self-assembled spheres as a hard template^20^,^21^,^22^) priorly deposited in the channels of the porous membrane. Compared to the hard/soft-templating method, the hard/hard-templating method based on 1D porous membrane and self-assembled nanospheres (<50 nm) would have great advantage in producing nanoporous metal nanowires with controlled large mesopores (>10 nm), which are important for efficient diffusion of guest species during the reactions. The nanospheres (e.g., silica nanospheres) used to fill in the porous membrane can be facilely prepared by the published method^23^,^24^ and their easily controlled diameters of 10–50 nm can define the mesopores of the metals^25^. Also, the long-range order of the mesoporous structures in the final metal replicas can be obtained from the close-packed nanospheres with high thermal stability.

Li *et al*.^20^ first reported the preparation of porous metal (Au and Ni) wires with controllable morphology from directed assemblies of spheres in 1D porous membranes, but the macroscale of the pores limits their applications. Bechelany *et al*.^22^ reported a similar dual templating method to fabricate Co nanowires with controlled porosity. However, in the previous reports, the systematic study of the fabrication of metal nanowires with various large mesopores (10–50 nm) and their properties has not been described. Furthermore, electrochemical deposition was typically used to grow the metals inside the dual templates in these reports. By contrast, chemical reduction deposition offers a simple and cost-effective way to replicate the structure of the porous template in the whole thickness range, without the need of using external electric field sources and electronically conductive substrates^26^,^27^.

In this study, we first report the chemical reduction fabrication of the mesoporous Pt nanowires with ordered large mesopores from AAO membranes with silica nanospheres assembled in the channels. Also, the electrocatalytic properties of the mesoporous Pt nanowires for the methanol oxidation reaction (MOR) are investigated. The Pt nanowires with ordered large mesopores would be desirable to improve the electrocatalytic activity due to the efficient transport of molecules and ions from the interconnected ordered large mesopores for increasing accessibility to the active sites, as well as enhance the durability owing to their longitudinal axis structures.

## Results

**Figure 1:**
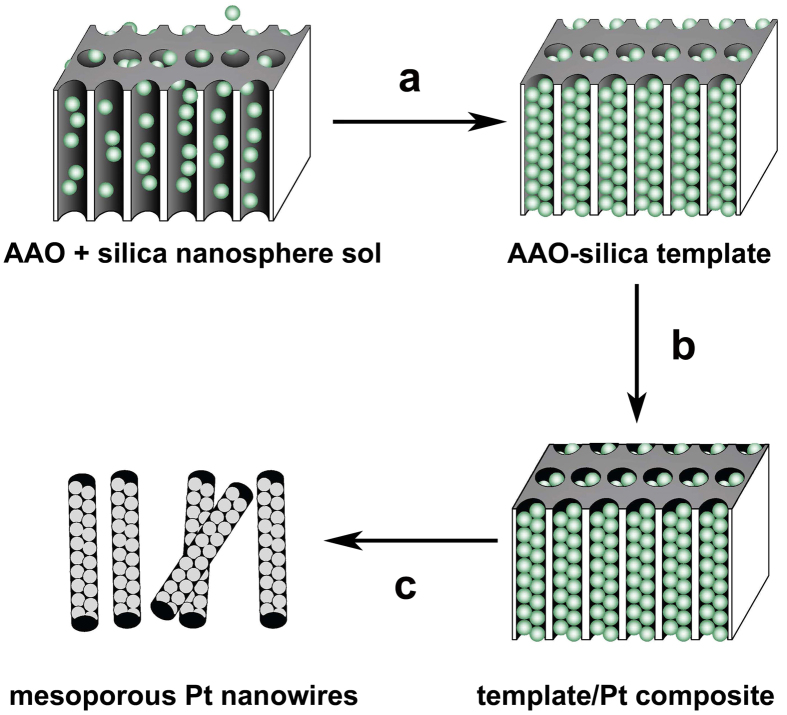
Schematic of the fabrication of the ordered mesoporous Pt nanowires. (**a**) Silica nanospheres are packed in the channels of the AAO membrane; (**b**) Pt is deposited in the void space of the AAO-silica template; (**c**) AAO-silica template is removed to produce the ordered mesoporous Pt nanowires.

Figure 1 illustrates the synthetic procedure for the preparation of the Pt nanowires with large mesopores (see Methods for more details). First, silica nanospheres were self-assembled in the channels of the AAO membrane to form the AAO-silica composite. Then Pt was deposited in the void space of the AAO-silica composite by chemical reduction deposition method. Subsequent removal of the AAO membrane and silica nanospheres with a dilute HF solution produced the freestanding mesoporous Pt nanowires. In order to obtain well-defined interconnected mesoporous structure over the whole nanowire length, the ratio of the AAO pore size to silica nanosphere diameter was selected larger than 4. In this work, we prepare the mesoporous Pt nanowires produced from 15, 30 and 45 nm silica nanospheres in 200 nm pore AAO membranes (abbreviated as 15/200-Pt, 30/200-Pt and 45/200-Pt nanowires, respectively).

**Figure 2:**
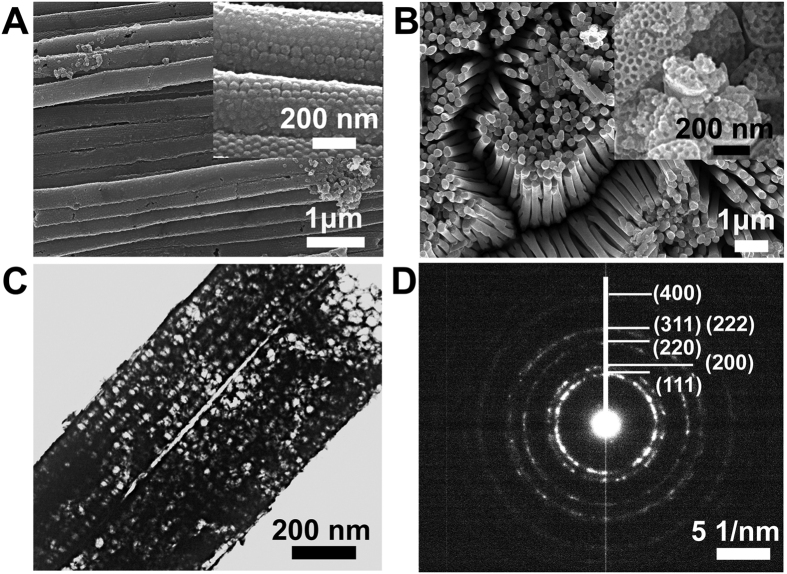
Structural characterization of the mesoporous Pt nanowires. (**A**) SEM image of the nanowires made of 45-nm silica nanospheres; inset: higher-magnification SEM image, (**B**) SEM image of the mesoporous Pt nanowires made from 200-nm pore AAO and 45 nm silica nanospheres; inset: higher-magnification SEM image, (**C**) the corresponding TEM image, and (**D**) the corresponding SAED pattern.

Figure 2A shows a typical scanning electron microscopy (SEM) image of the nanowires composed of 45-nm diameter silica nanospheres, obtained by etching away the AAO template from the AAO-silica composite using a phosphoric acid solution. The silica nanospheres are arranged in a hexagonal close-packing, which is essential for the formation of ordered mesoporous Pt nanowires. Figure 2B reveals the SEM image of the 45/200-Pt nanowires. The average diameter of the Pt nanowires is ca. 300 nm that disagrees with the surface pore size (~200 nm) of the AAO membrane, because the diameter of the inside channels of the AAO membrane is larger than that of the surface pores. Ordered spherical mesopores can be clearly seen from the inset of Fig. 2B. The porous Pt nanowires exhibit well-ordered mesoporous structure and the mesopore size is ca. 45 nm that is consistent with the particle size of the original silica nanospheres, indicating negligible shrinkage of the metallic structure during removal of the silica template. The corresponding transmission electron microscopy (TEM) image of the 45/200-Pt nanowires with ordered mesopores is shown in Fig. 2C. The interconnected ordered mesoporous structure is important for the facile diffusion of reactants during the electrocatalytic electrode reactions. The selected area electron diffraction (SAED) pattern exhibits polycrystalline face-centered cubic (fcc) Pt feature with homogenous diffraction rings (Fig. 2D), which corresponds to the presence of many small Pt nanoparticles in the mesopore walls. The detailed structural characterization of the 15/200-Pt nanowires is presented in Fig. S1 (Supplementary Information), also revealing the well-ordered mesoporous structure of the Pt nanowires.

**Figure 3:**
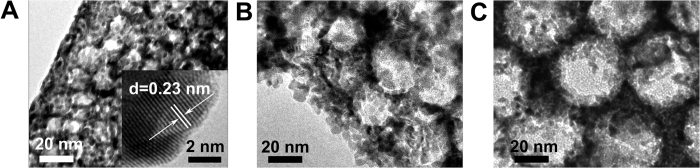
TEM micrographs of the mesoporous Pt nanowires. High-magnification TEM images of the mesoporous Pt nanowires with (**A**) 15 nm mesopores (inset: high-resolution image showing the lattice fringes), (**B**) 30 nm mesopores and (**C**) 45 nm mesopores prepared from the 200-nm AAO-silica templates.

Figure 3A–C show highly magnified TEM images of the 15/200-Pt, 30/200-Pt and 45/200-Pt nanowires, respectively. The high resolution TEM (HR-TEM) image (inset of Fig. 3A) reveals the lattice fringes with a spacing of 0.23 nm in the mesopore walls, which is in agreement with the d-spacing of adjacent (111) crystallographic planes in face-centered cubic (fcc) Pt, indicating a high crystallinity of the Pt nanoparticles. These mesoporous Pt nanowires have the same diameter, but with different mesopore sizes. As can be seen in the images, the mesopore walls are composed of small Pt nanoparticles. With the decrease of the mesopore size, the number of Pt nanoparticles aggregated in the mesopore walls reduces.

**Figure 4:**
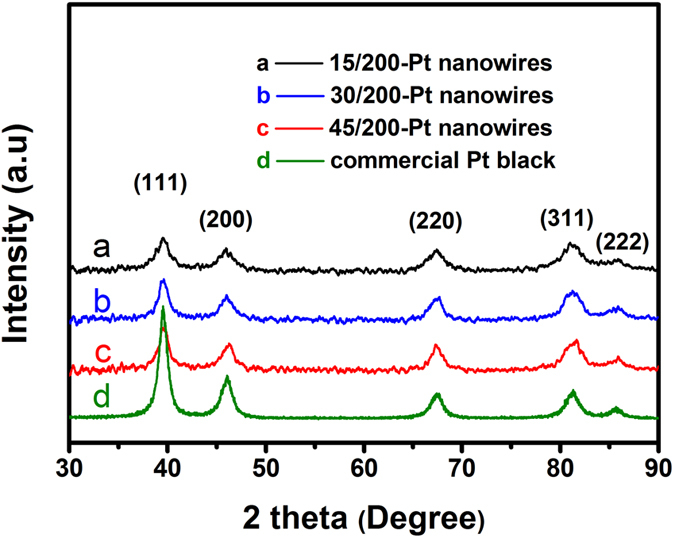
XRD patterns of the mesoporous Pt nanowires. XRD patterns of (**a**) 15/200-Pt nanowires, (**b**) 30/200-Pt nanowires, (**c**) 45/200-Pt nanowires, and (**d**) commercial Pt black.

Powder X-ray diffraction (XRD) was employed to further investigate the Pt particle size of the catalysts (Fig. 4). The Pt nanoparticle sizes of the 15/200-Pt, 30/200-Pt and 45/200-Pt nanowires, as well as the commercial Pt black catalyst estimated by the Scherrer equation are 3.8 nm, 4.9 nm, 5.2 nm, and 5.5 nm, respectively. In overall trend, the Pt particle size increases with the mesopore size of the Pt nanowires.

**Figure 5:**
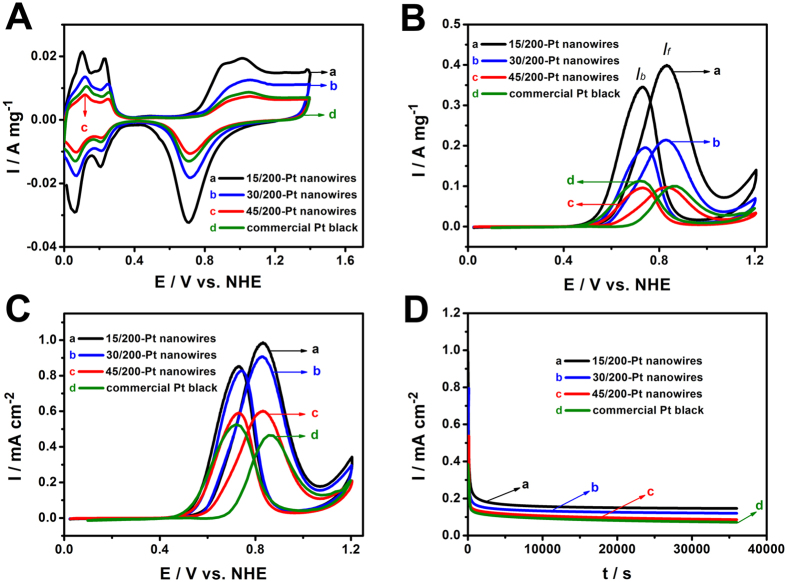
Comparison of the electrocatalytic properties of different mesoporous Pt nanowires. (**A**) Cyclic voltammograms (CVs) of the (a) 15/200-Pt nanowires, (b) 30/200-Pt nanowires, (c) 45/200-Pt nanowires, and (d) commercial Pt black catalyst in 0.5 M H_2_SO_4_. (**B**) CVs of the catalysts in 0.5 M H_2_SO_4_ and 1 M CH_3_OH. (**C**) ECSA-normalized CVs of the catalysts in 0.5 M H_2_SO_4_ and 1 M CH_3_OH. (**D**) Chronoamperometric curves for the catalysts in 0.5 M H_2_SO_4_ and 1 M CH_3_OH at 0.8 V. The scan rates of the CVs are 50 mV s^−1^.

Figure 5 compares the MOR performances of the mesoporous Pt nanowires and the commercial Pt black catalyst. The electrocatalytic results of the four catalysts are summarized in Table 1. Figure 5A reveals the cyclic voltammograms (CVs) in 0.5 M H_2_SO_4_ solution at a scan rate of 50 mV s^−1^. The specific electrochemically active surface area (ECSA) of Pt was estimated from the area of desorption of atomic hydrogen on the curve of the CV between 0 and 0.35 V by the following equation:^28^,^29^


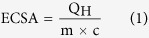


where Q_H_ is the total charge (mC cm^−2^), m is actual Pt loading (mg cm^−2^) on the GC substrate, and c represents the charge required to oxidize a monolayer of hydrogen on a Pt surface (0.21 mC cm^−2^). The ECSA of the 15/200-Pt nanowires is calculated to be 40.5 m^2^ g^−1^, which is much larger than that of the 30/200-Pt nanowires (23.6 m^2^ g^−1^), 45/200-Pt nanowires (16.2 m^2^ g^−1^) and commercial Pt black (21.3 m^2^ g^−1^), as well as higher than or comparable to that of the reported 1D nanostructured Pt (e.g., porous dendritic Pt nanotubes (23.3 m^2^ g^−1^)^30^, multigrain Pt nanowires on sulfur-doped graphene (24.5 m^2^ g^−1^)^31^, Pt nanowire arrays (25.4 m^2^ g^−1^)^32^, Pt nanowire arrays on sulfur doped graphene (40 m^2^ g^−1^)^33^ and the mesoporous Pt materials (e.g., mesoporous Pt nanospheres (18.0 m^2^ g^−1^)^13^, 3D macro-/mesoporous Pt (38.3 m^2^ g^−1^)^12^, and mesoporous Pt nanoparticles (42.6 m^2^ g^−1^)^34^). The larger ECSA of the 15/200-Pt nanowires may arise from the smaller mesopore size which can facilitate the formation of smaller Pt particles and improve the Pt particle dispersion. Because the agglomeration of Pt particles on the framework is more severe with the increasing of the pore size of Pt nanowires, the 45/200-Pt nanowires have smaller ECSA than the commercial Pt black even though the former are composed of smaller Pt particles than the latter.

The electrochemical performances of these catalysts for MOR were conducted on CV testing. The CVs obtained in 0.5 M H_2_SO_4_ and 1 M CH_3_OH solution at a scan rate of 50 mV s^−1^ are shown in Fig. 5B. In the forward scan, the peak current densities are 398, 213, 98 and 101 mA mg^−1^ for the reactions on the 15/200-Pt, 30/200-Pt and 45/200-Pt nanowire, and commercial Pt black electrodes, respectively. The highest methanol oxidation mass activity of the 15/200-Pt nanowires could be attributed to the greatly increased ECSA. Since the mass-normalized activity is an important parameter to evaluate the activity of surface atoms and the Pt utilization, the 15/200-Pt nanowires show the best utilization efficiency of Pt which is essential for the fuel cell applications. After normalization to the ECSAs (Fig. 5C), the 15/200-Pt nanowires still have the highest methanol oxidation activity, which may be attributed to the smallest interconnected mesopores resulting in highly dispersed Pt nanoparticles^17^. In addition, the linear sweep voltammograms (LSVs) and the corresponding Tafel plots (Supplementary Fig. S2) also suggest that the 15/200-Pt nanowire catalyst is promising as a potential practical electrocatalyst for methanol oxidation.

Since the agglomeration of Pt particles on the mesopore walls becomes more severe with the increase of the pore size of Pt nanowires, the 45/200-Pt nanowires exhibit smaller ECSA and lower mass activity than the commercial Pt black catalyst even though the Pt particle size of the 45/200-Pt nanowires is a bit smaller than that of the commercial Pt black catalyst. However, the 45/200-Pt nanowires have improved specific activity compared with the Pt black catalyst, which might arise from the 1D structure that could facilitate the reaction kinetics and improve the diffusion of reactants due to the path directing effects of the structural anisotropy^35^,^36^.

From Fig. 5B, the ratio of *I*_*f*_ (the peak current of the forward scan) to *I*_*b*_ (the peak current of the backward scan) of the 15/200-Pt nanowires catalyst is 1.15, which is higher than that of the 30/200-Pt nanowires (1.08), 45/200-Pt nanowires (1.02), and commercial Pt black (0.90). A higher *I*_*f*_/*I*_*b*_ is indicative of better tolerance for poisoning of carbonaceous species^37^. The *I*_*f*_/*I*_*b*_ value of the 15/200-Pt nanowire catalyst is comparable to that of the reported mesoporous Pt nanoparticles (1.07)^34^, macro-/mesoporous Pt (1.25)^12^, and mesoporous Pt nanorods (1.25)^16^.

The chronoamperomograms (Fig. 5D) recorded at 0.8 V indicated that the current density of the 15/200-Pt nanowires was higher than that of the 30/200-Pt and 45/200-Pt nanowires, and the commercial Pt black catalyst over the entire time range, also demonstrating the improved electrocatalytic performance of the 15/200-Pt nanowires for the MOR. In addition, the long-term poisoning rate (δ) is calculated by measuring the linear decay of the current for a period of more than 5000 s from Fig. 5D by using the following equation:^38^,^39^


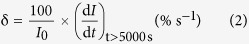


where (d*I*/d*t*)_t > 5000 s_ is the slope of the linear portion of current decay and *I*_0_ is the current at the start of polarization back extrapolated from the linear current decay. The poisoning rates are calculated to be 0.00028, 0.00054, 0.00079 and 0.00061% s^−1^ for the 15/200-Pt, 30/200-Pt and 45/200-Pt nanowires, and the commercial Pt black catalyst, respectively. The result reveals that, compared with the commercial Pt black, 15/200-Pt and 30/200-Pt nanowires have better poisoning tolerance and stability, which may result from the combination of the advantages of mesoporous structure that improves the dispersion of Pt nanoparticles and one-dimensional architecture that makes the Pt nanowires less vulnerable to dissolution, Ostwald ripening, and aggregation^19^,^30^. An increase in the mesopore size of Pt nanowires results in a decrease in the anti-poisoning performance and stability, which may arise from the more severe aggregation of Pt particles in the mesopore walls (Fig. 3A–C) that deteriorates the mass transfer efficiency and makes the Pt particles grow rapidly in the long-time reaction. The 45/200-Pt nanowires show better poisoning tolerance in the first CV curve but worse stability in the long-time reaction than the commercial Pt black, which is probably attributed to that the agglomerated Pt nanoparticles on the mesopore walls tend to grow faster than the dispersed Pt nanoparticles of the commercial Pt black.

## Discussion

In summary, mesoporous Pt nanowires with ordered uniform large mesopores (15 nm, 30 nm, and 45 nm) have been successfully synthesized by chemical reduction deposition method from a dual-templating technique using the porous AAO membranes with silica nanospheres self-assembled in the channels as the templates. The mesopore walls of the Pt nanowires are composed of polycrystalline Pt nanoparticles, and as the mesopore size increases, more Pt nanoparticles aggregate in the mesopore walls. Overall, the prepared mesoporous Pt nanowires exhibit enhanced electrocatalytic activity, and improved poisoning tolerance and durability for the methanol oxidation reaction (MOR) compared with the commercial Pt black catalyst, which may be related to the interconnected ordered mesopores that allow the facile transport of the reactant and product molecules and ions to and from the catalyst and the 1D structure that could facilitate the reaction kinetics as well as improve the diffusion of reactants. Due to the smaller and more dispersed Pt nanoparticles in the mesopore walls, the mesoporous Pt nanowires with 15 nm mesopores have better MOR performance than the other mesoporous Pt nanowires. It is expected that the present method could be extended to prepare other ordered mesoporous metal (e.g., Ni, and Pd) or alloy (e.g., Pt-Ni, and Pt-Ru alloys) nanowires, which may be of technological importance in various fields, such as nanodevices, fuel cells, and catalysis.

## Methods

### Materials

Tetraethyl orthosilicate (TEOS, 98%), L-lysine (≥98%), H_2_PtCl_6_·6H_2_O (≥37.5% Pt basis), H_3_PO_4_ (≥85 wt.% in H_2_O) and ethanol (99.7%) were obtained from Beijing Chemical Reagents Company. Dimethylamine borane (DMAB) ((CH_3_)_2_NHBH_3_, 97%), and Nafion solution (5 wt.%) were purchased from Sigma-Aldrich. TEOS was freshly vacuum-distilled before used. The other chemicals were used as received without further purification. The 200 nm-pore AAO membranes with channel diameter between 200 and 400 nm were purchased from Whatman International Ltd. The commercial Pt Black (HiSpec1000) was obtained from Johnson Matthey Company, Ltd.

### Synthesis of silica nanospheres

The silica nanospheres of about 15, 30, and 45 nm diameters were prepared by hydrolysis of TEOS in the presence of L-lysine^11^. To fabricate the silica nanospheres with diameter of ~15 nm, 0.19 g of L-lysine, 12.00 g of ethanol and 180 mL of water were mixed in a three-necked flask and stirred for 20 min. Then the flask was transferred into an oil bath and stirred at 90 °C, after which 13.50 g of TEOS was added under stirring at 500 rpm. The silica nanosphere (~15 nm in diameter) sol was obtained after continuous magnetic stirring for 48 h. The silica nanospheres of ~30 nm diameter were fabricated through a seeded growth approach with the ~15 nm nanospheres used as seeds. Subsequent repeated addition of TEOS (each time 27.00 g), followed by stirring (500 rpm) at 90 °C for 48 h, was carried out twice to increase the particle size to ~30 nm. The ~45 nm silica nanospheres were produced following the same steps using the ~15 nm nanospheres as seeds, but each repeated addition of TEOS is 47.25 g in mass.

### Synthesis of AAO-silica template

To get the AAO-silica template, a piece of AAO membrane was put in the bottom of a 50 mL beaker containing 10 mL silica nanosphere sol (5 wt.%), and then the beaker was placed in 40 °C oven. After evaporation the solvent for 2 days, the silica nanospheres were self-assembled in the channels of the AAO membrane by the combined action of capillary, gravitational and electrostatic forces. Then the material was heated at 550 °C for 5 h at a heating rate of 1 °C/min to produce the robust AAO-silica templates. Strands of nanowires composed of silica nanospheres can be obtained by etching away the AAO template from the AAO-silica composite using dilute H_3_PO_4_ solution (10 wt.%).

### Synthesis of mesoporous metal nanowires from AAO-silica template

To prepare the mesoporous Pt nanowires, a piece of AAO-silica template was first immersed in the precursor solution containing H_2_PtCl_6_·H_2_O and C_2_H_5_OH (1:1 in mass ratio) for 1 h. Then, the template was removed from the solution and dried at room temperature. After that, the sample was reduced by the vapor of the dimethylamine borane (DMAB) at room temperature for 24 h in a closed vial^18^. Finally, the mesoporous Pt nanowires were obtained by removing the AAO-silica template with a 5 wt.% HF solution (24 h).

### Characterization

Scanning electron microscopy (SEM) images were obtained on a Hitachi S-4700 FEG scanning electron microscope. Transmission electron microscopy (TEM) and high-resolution TEM (HR-TEM) were carried out on a Hitachi H800 and a JEOL JEM-3010 transmission electron microscope, respectively (both operating at 200 kV). Powder X-ray diffraction (XRD) data were collected on a Shimadzu XRD-6000 diffractometer with Cu Kα radiation (λ = 1.5418 Å).

### Electrochemical experiments

Cyclic voltammetry (CV) and chronoamperometry measurements were carried out in a potentiostat/galvanostat (Reference 600, Gamry Instruments) using a conventional three-electrode cell consisting of a glassy carbon (GC) electrode (5 mm in diameter) as the work electrode, a double junction Ag/AgCl (saturated KCl) electrode as the reference electrode, and a Pt flag as the counter electrode. All electrode potentials were measured against the Ag/AgCl reference electrode and converted to the normal hydrogen electrode (NHE) potentials by using relationship of E_NHE_ = E_Ag/AgCl; sat. KCl_ + 0.197 V. To prepare the working electrode, the GC electrode was polished to a mirror finish using 50 nm alumina suspensions, and cleaned by ultrasonication in nitric acid, ethanol, and deionized water. The catalyst (5 mg) was ultrasonically dispersed in 5 mL ethanol by ultrasonication for 1 h to obtain a 1.0 mg mL^−1^ homogeneous ink-like suspension. A volume of 5 μL of the suspension was then pipetted onto the GC substrate. After drying at room temperature, 5 μL of Nafion solution (5 wt.%) was pipetted on the GC substrate and dried completely. The CV tests were performed in argon-purged 0.5 M H_2_SO_4_ solution with or without 1 M CH_3_OH at room temperature.

## Additional Information

**How to cite this article**: Zhang, C. *et al*. Controlled Synthesis of Pt Nanowires with Ordered Large Mesopores for Methanol Oxidation Reaction. *Sci. Rep*. **6**, 31440; doi: 10.1038/srep31440 (2016).

## Supplementary Material

Supplementary Information

## Figures and Tables

**Table 1:** Pt particle sizes, ECSAs, peak current densities for MOR in the forward scan, and *I*_*f*_/*I*_*b*_ of the catalysts.

Sample	Pt particlesize^a^ (nm)	ECSA(m^2^ g^−1^)	Peak current density for MOR		*I*_*f*_/*I*_*b*_
			Mass activity(mA mg^−1^)	Specific activity(mA cm^−2^)	
15/200-Pt nanowires	3.8	40.5	398	0.98	1.15
30/200-Pt nanowires	4.9	23.6	213	0.90	1.08
45/200-Pt nanowires	5.2	16.2	98	0.60	1.02
commercial Pt black	5.5	21.3	101	0.47	0.90

^a^The data were calculated by XRD.

## References

[b1] Feng Y., Yang J., Liu H., Ye F. & Yang J. Selective electrocatalysts toward a prototype of the membraneless direct methanol fuel cell. Sci. Rep. 4, 580–580 (2014). 10.1038/srep03813 PMC3897953 24448514

[b2] Li X. & Faghri A. Review and advances of direct methanol fuel cells (DMFCs) part I: Design, fabrication, and testing with high concentration methanol solutions. J. Power Sources 226, 223–240 (2013).

[b3] Górzny M. Ł., Walton A. S. & Evans S. D. Synthesis of High-Surface-Area Platinum Nanotubes Using a Viral Template. Adv. Funct. Mater. 20, 1295–1300 (2010).

[b4] Li Z., Zhang L., Huang X., Ye L. & Lin S. Shape-controlled synthesis of Pt nanoparticles via integration of graphene and β-cyclodextrin and using as a noval electrocatalyst for methanol oxidation. Electrochim. Acta 121, 215–222 (2014).

[b5] Do I. & Drzal L. T. Ionic Liquid-Assisted Synthesis of Pt Nanoparticles onto Exfoliated Graphite Nanoplatelets for Fuel Cells. ACS Appl. Mat. Inter. 6, 12126–12136 (2014). 10.1021/am5012832 25036977

[b6] Tao K. . Short peptide-directed synthesis of one-dimensional platinum nanostructures with controllable morphologies. Sci. Rep. 3, 1–6 (2013). 10.1038/srep02565 PMC3759059 23995118

[b7] Choi S. M., Kim J. H., Ju Y. J., Yoon E. Y. & Kim W. B. Pt nanowires prepared via a polymer template method: Its promise toward high Pt-loaded electrocatalysts for methanol oxidation. Electrochim. Acta 53, 5804–5811 (2008).

[b8] Ci S., Zou J., Zeng G., Luo S. & Wen Z. Single crystalline Pt nanotubes with superior electrocatalytic stability. J. Mater. Chem. 22, 16732–16737 (2012).

[b9] Xiao Y. . Preparation of Pt hollow nanotubes with adjustable diameters for methanol electrooxidation. Rsc Adv. 4, 21176–21179 (2014).

[b10] Yang M. Monodispersed hollow platinum nanospheres: facile synthesis and their enhanced electrocatalysis for methanol oxidation. J. Mater. Chem. A 2, 13738–13743 (2014).

[b11] Han-Pu L. . Pt hollow nanospheres: facile synthesis and enhanced electrocatalysts. Angew. Chem. Int. Ed. 43, 1540–1543 (2004). 10.1002/anie.200352956 15022227

[b12] Zhang C. . Synthesis of three-dimensionally ordered macro-/mesoporous Pt with high electrocatalytic activity by a dual-templating approach. J. Power Sources 245, 579–582 (2014).

[b13] Chen P. K. . New Synthesis of MCM-48 Nanospheres and Facile Replication to Mesoporous Platinum Nanospheres as Highly Active Electrocatalysts for the Oxygen Reduction Reaction. Chem. Mater. 25, 4269–4277 (2013).

[b14] Bi Y. & Lu G. Control growth of uniform platinum nanotubes and their catalytic properties for methanol electrooxidation. Electrochem. Commun. 11, 45–49 (2009).

[b15] Chen Z., Waje M., Li W. & Yan Y. Supportless Pt and PtPd nanotubes as electrocatalysts for oxygen-reduction reactions. Angew. Chem. Int. Ed. 46, 4060–4063 (2007). 10.1002/anie.200700894 17476642

[b16] Li C., Takaaki S. & Yusuke Y. Electrochemical synthesis of one-dimensional mesoporous Pt nanorods using the assembly of surfactant micelles in confined space. Angew. Chem. Int. Ed. 52, 8208–8211 (2013). 10.1002/anie.201303035 23804435

[b17] Zhong Y., Xu C. L., Kong L. B. & Li H. L. Synthesis and high catalytic properties of mesoporous Pt nanowire array by novel conjunct template method. Appl. Surf. Sci. 255, 3388–3393 (2008).

[b18] Yusuke Y., Azusa T., Tomota N., Satoru I. & Kazuyuki K. Pt fibers with stacked donut-like mesospace by assembling pt nanoparticles: guided deposition in physically confined self-assembly of surfactants. J. Am. Chem. Soc. 130, 5426–5427 (2008). 18370388 10.1021/ja800269c

[b19] Takai A., Yamauchi Y. & Kuroda K. Fabrication of mesoporous Pt nanotubes utilizing dual templates under a reduced pressure condition. Chem. Commun. 35, 4171–4173 (2008). 10.1039/b804072a 18802519

[b20] Li F., He J., Zhou W. L. & Wiley J. B. Synthesis of porous wires from directed assemblies of nanospheres. J. Am. Chem. Soc. 125, 16166–16167 (2003). 14692739 10.1021/ja038452+

[b21] Ahn H. J. & Wang D. Synthesis and electrochemical properties of porous Pt wire electrodes for methanol electro-oxidation. Solid State Sci. 13, 1612–1615 (2011).

[b22] Bechelany M. . Nanowires with controlled porosity for hydrogen production. J. Mater. Chem. A 1, 2133–2138 (2013).

[b23] Wei F. . Hierarchical nanofabrication of microporous crystals with ordered mesoporosity. Nat. Mater. 7, 984–991 (2008). 18953343 10.1038/nmat2302

[b24] Snyder M. A., Alex J. L., Davis T. M., Scriven L. E. & Michael T. Silica nanoparticle crystals and ordered coatings using lys-sil and a novel coating device. Langmuir 23, 9924–9928 (2007). 17625899 10.1021/la701063v

[b25] Yoshiyuki K., Yusuke Y. & Kazuyuki K. Integrated structural control of cage-type mesoporous platinum possessing both tunable large mesopores and variable surface structures by block copolymer-assisted Pt deposition in a hard-template. Chem. Commun. 46, 1827–1829 (2010). 10.1039/b921016d 20198222

[b26] Hulteen J. C. & Martin C. R. A general template-based method for the preparation of nanomaterials. J. Mater. Chem. 7, 1075–1087 (1997).

[b27] Zhang C. . Template-assisted chemical synthesis of Au opal photonic crystal film with complete photonic band gaps in the visible. Mater. Lett. 131, 272–275 (2014).

[b28] Gasteiger H. A., Kocha S. S., Sompalli B. & Wagner F. T. Activity benchmarks and requirements for Pt, Pt-alloy, and non-Pt oxygen reduction catalysts for PEMFCs. Appl. Catal. B-Environ 56, 9–35 (2005).

[b29] Ralph T. R. . ChemInform Abstract: Low Cost Electrodes for Proton Exchange Membrane Fuel Cells. Performance in Single Cells and Ballard Stacks. Cheminform 144, 3845–3857 (1996).

[b30] Zhang G. . Porous Dendritic Platinum Nanotubes with Extremely High Activity and Stability for Oxygen Reduction Reaction. Sci. Rep. 3, 776–776 (2013). 10.1038/srep01526 PMC3607176 23524665

[b31] Hoque M. A. . Electrocatalysts: Multigrain Platinum Nanowires Consisting of Oriented Nanoparticles Anchored on Sulfur-Doped Graphene as a Highly Active and Durable Oxygen Reduction Electrocatalyst. Adv. Mater. 27, 1229–1234 (2015). 25417577 10.1002/adma.201404426

[b32] Du S. . Plasma nitriding induced growth of Pt-nanowire arrays as high performance electrocatalysts for fuel cells. Sci. Rep. 4, 6439–6439 (2014). 25241800 10.1038/srep06439 PMC4170194

[b33] Wang R. . Controlled growth of platinum nanowire arrays on sulfur doped graphene as high performance electrocatalyst. Sci. Rep. 3, 2431–2431 (2013). 23942256 10.1038/srep02431 PMC3743054

[b34] Liang W. & Yusuke Y. Synthesis of mesoporous Pt nanoparticles with uniform particle size from aqueous surfactant solutions toward highly active electrocatalysts. Chem.-Eur. J. 17, 8810–8815 (2011). 21732437 10.1002/chem.201100386

[b35] Sun S. . A Highly Durable Pt Nanocatalyst for Proton Exchange Membrane Fuel Cells: the Multiarmed Star-like Nanowire Single Crystal. Angew. Chem. Int. Ed. 50, 422–426 (2010). 10.1002/anie.201004631 21082633

[b36] Zhang X. . Porous platinum nanowire arrays for direct ethanol fuel cell applications. Chem. Commun. 2, 195–197 (2009). 10.1039/b813830c 19099066

[b37] Xu C. . Nanotubular Mesoporous Bimetallic Nanostructures with Enhanced Electrocatalytic Performance. Adv. Mater. 21, 2165–2169 (2009).

[b38] Jiang J. & Kucernak A. Electrooxidation of small organic molecules on mesoporous precious metal catalysts I: CO and methanol on platinum. J. Electroanal. Chem. 533, 153–165 (2002).

[b39] Jiang J. & Kucernak A. Electrooxidation of small organic molecules on mesoporous precious metal catalysts : II: CO and methanol on platinum–ruthenium alloy. J. Electroanal. Chem. 543, 187–199 (2003).

