# Supplementary material for: Controlled Synthesis of Pt Nanowires with Ordered Large Mesopores for Methanol Oxidation Reaction
Source: Sci Rep. 2016 Aug 23;6:31440. doi: 10.1038/srep31440 (PMC4994005; doi:10.1038/srep31440)
Supplement: Supplementary Information [file srep31440-s1.pdf]

Supporting Information

Controlled Synthesis of Pt Nanowires with Ordered Large Mesopores for Methanol Oxidation Reaction

Chengwei Zhang[1,2], Lianbin Xu[1*], Yushan Yan[3] & Jianfeng Chen[1]

[1]State Key Laboratory of Organic−Inorganic Composites, Beijing University of Chemical Technology, Beijing 100029, China

[2]Tianjin Key Laboratory of Materials Laminating Fabrication and Interface Control Technology, Hebei University of Technology, Tianjin 300130, China

[3]Department of Chemical and Biomolecular Engineering, University of Delaware, Newark, Delaware 19716, USA

Corresponding author. Tel: +86-10-64449241; fax: +86-10-64434784.

E-mail: xulb@mail.buct.edu.cn

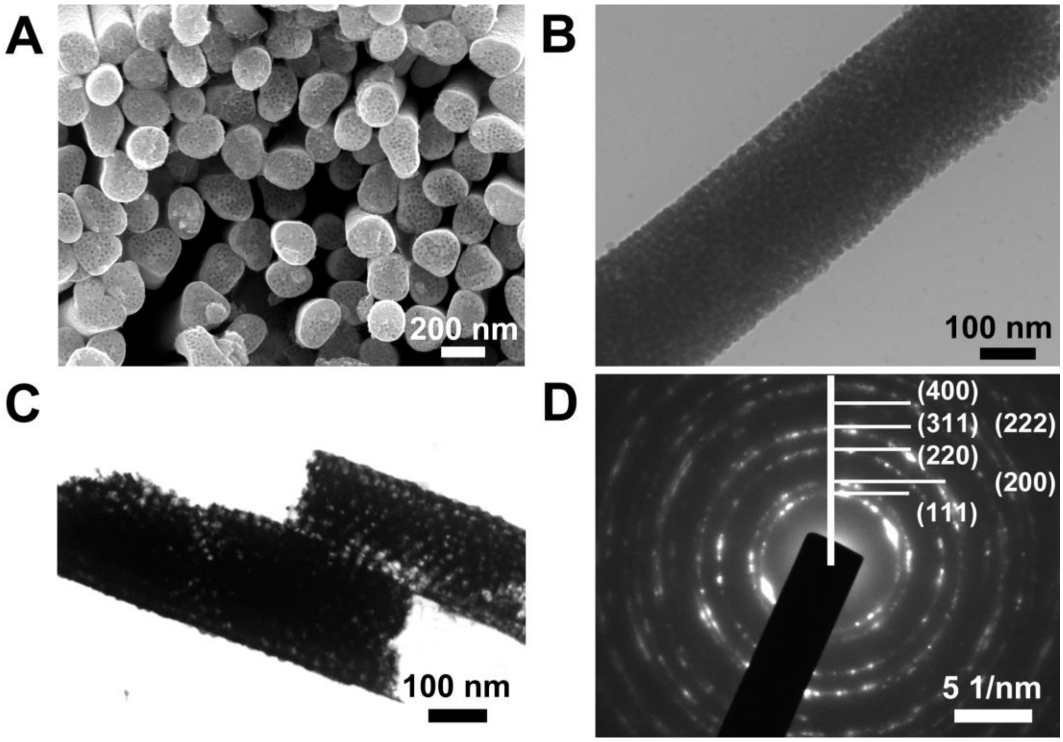

Figure S1. Structural characterization of mesoporous Pt nanowires. (A) SEM image of the 15/200-Pt nanowire array, (B) TEM image of one nanowire made of 15-nm silica nanospheres, (C) TEM image of the 15/200-Pt nanowires, and (D) the corresponding SAED pattern of the 15/200-Pt nanowires.

Figure S1A shows a typical SEM image of the 15/200-Pt nanowires with 15-nm mesopores. Figure S1B is the TEM image of the silica sphere nanowires, which was obtained by etching away the AAO template from the AAO-silica sphere composite. Figure S1C reveals that the 15/200-Pt nanowires have ordered mesoporous structure and the mesopore size is ca. 15 nm that agrees with the diameter of the silica nanospheres in Fig. S1B. Figure S1D is the SAED pattern of the 15/200-Pt nanowires, which are composed of polycrystalline Pt nanoparticles.

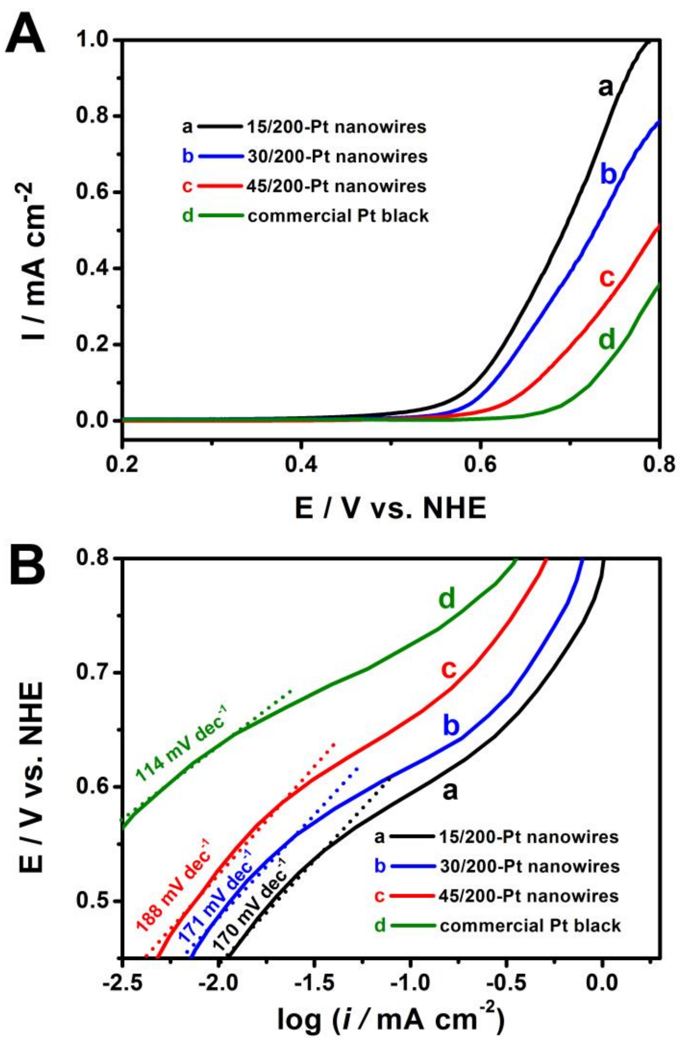

Figure S2. Comparison of the electrocatalytic properties of different mesoporous Pt nanowires. (A) Linear sweep voltammograms (LSVs) of the (a) 15/200-Pt nanowires, (b) 30/200-Pt nanowires, (c) 45/200-Pt nanowires, and (d) commercial Pt black catalyst in 0.5 M H_2SO_4 and 1 M CH_3OH solution at a scan rate of 1 mV s^{-1}, and (B) the corresponding Tafel plots.

To further investigate the electrocatalytic properties and MOR kinetics of the mesoporous Pt nanowires, the linear sweep voltammograms (LSVs) were measured in Ar-saturated 0.5 M H_2SO_4 and 1 M CH_3OH solution at a scan rate of 1 mV s^{-1}. To properly measure and compare the onset potential values of the catalysts for MOR, a minimum value of specific current density is fixed. All onset potentials of the Pt catalysts obtained from LSV curves are taken as 0.02 mA cm^{-2}. As shown in Fig. S2A, the onset potential of the 15/200-Pt nanowire catalyst occurs at about 0.50 V, which is substantially more negative than that of the 30/200-Pt nanowire (0.54 V), 45/200-Pt

nanowire (0.58 V) and commercial Pt black (0.66 V) catalysts. The low onset potential presents clear evidence for the excellent electrocatalytic MOR activity.

Figure S2B presents the Tafel plots derived from the LSVs. We used the LSVs recorded at 1 mV s^{-1} to obtain the Tafel plots, which are close to steady-state polarization curves. From Fig. S2B, it can be observed that the Pt catalysts present two nearly linear regions which intersect at approximately 0.65 V for the reaction, indicating a change in the rate determining step. In the region below 0.65 V, the Tafel slope of MOR on the commercial Pt black catalyst is 114 mV dec^{-1}, which is a deviation from the theoretical value 120 mV dec^{-1} predicted for one-electron transfer reaction as rate determining step. However, the Tafel slopes for the mesoporous Pt nanowires in the same region are much higher, (170 mV dec^{-1} for 15/200-Pt nanowires, 171 mV dec^{-1} for 30/200-Pt nanowires, and 188 mV dec^{-1} for 45/200-Pt nanowires), indicating a faster methanol dehydrogenation even at the relatively low overpotential region[1,2]. In addition, from the Tafel plots, the 15/200-Pt nanowire catalyst exhibit the current density of 0.120 mA cm^{-2} at 0.60 V vs NHE (potential near to the working potential in a DMFC), which is much higher than that of the other Pt nanowires (0.064 mA cm^{-2} for 30/200-Pt nanowires and 0.028 mA cm^{-2} for 45/200-Pt nanowires) and the commercial Pt black catalyst (0.005 mA cm^{-2}), indicating that the 15/200-Pt nanowire catalyst holds promise as a potential practical electrocatalyst for methanol oxidation.

References

1. Lu, J., Li, Y., Li, S. & Jiang, S. P. Self-assembled platinum nanoparticles on sulfonic acid-grafted graphene as effective electrocatalysts for methanol oxidation in direct methanol fuel cells. *Sci. Rep.* **6**, 1-12 (2016).
2. Bo, Z., Hu, D., Kong, J., Yan, J. & Cen, K. Performance of vertically oriented graphene supported platinum–ruthenium bimetallic catalyst for methanol oxidation. *J. Power Sources* **273**, 530-537 (2015).
